# Cost-effectiveness of infant respiratory syncytial virus preventive interventions in Mali: A modeling study to inform policy and investment decisions

**DOI:** 10.1016/j.vaccine.2021.06.086

**Published:** 2021-08-16

**Authors:** Rachel S. Laufer, Amanda J. Driscoll, Ranju Baral, Andrea G. Buchwald, James D. Campbell, Flanon Coulibaly, Fatoumata Diallo, Moussa Doumbia, Alison P. Galvani, Fadima C. Haidara, Karen L. Kotloff, Adama M. Keita, Kathleen M. Neuzil, Evan W. Orenstein, Lauren A.V. Orenstein, Clint Pecenka, Samba Sow, Milagritos D. Tapia, Justin R. Ortiz, Meagan C. Fitzpatrick

**Affiliations:** a Center for Vaccine Development & Global Health, 685 W. Baltimore St., University of Maryland School of Medicine, Baltimore, Maryland 21201, USA; b PATH, 2201 Westlake Avenue, Suite 200, Seattle, Washington 98121, USA; c Department of Environmental and Occupational Health, Colorado School of Public Health, 13001 East 17th Place Aurora, Colorado 80045, USA; d Centre pour le Développement des Vaccins, Ministère de la Santé, BP251 Bamako, Mali; e Center for Infectious Disease Modeling and Analysis, Yale School of Public Health, 135 College St., New Haven, CT 06510, USA; f Department of Pediatrics, Emory University School of Medicine, 1405 Clifton Rd, Atlanta, Georgia 30322, USA; g Department of Dermatology, Emory University School of Medicine, 1525 Clifton Rd, Atlanta, Georgia 30322, USA

**Keywords:** Global Health, Vaccine, Pediatric, Respiratory Syncytial Virus, Pneumonia, Prevention

## Abstract

**Importance:**

Low- and middle-income countries have a high burden of respiratory syncytial virus lower respiratory tract infections. A monoclonal antibody administered monthly is licensed to prevent these infections, but it is cost-prohibitive for most low- and middle-income countries. Long-acting monoclonal antibodies and maternal vaccines against respiratory syncytial virus are under development.

**Objective:**

We estimated the likelihood of respiratory syncytial virus preventive interventions (current monoclonal antibody, long-acting monoclonal antibody, and maternal vaccine) being cost-effective in Mali.

**Design:**

We modeled age-specific and season-specific risks of respiratory syncytial virus lower respiratory tract infections within monthly cohorts of infants from birth to six months. We parameterized with respiratory syncytial virus data from Malian cohort studies, as well as product efficacy from clinical trials. Integrating parameter uncertainty, we simulated health and economic outcomes for status quo without prevention, intra-seasonal monthly administration of licensed monoclonal antibody, pre-seasonal birth dose administration of a long-acting monoclonal antibody, and maternal vaccination. We then calculated the incremental cost-effectiveness ratio of each intervention compared to status quo from the perspectives of the government, donor, and society.

**Results:**

At a price of $3 per dose and from the societal perspective, current monoclonal antibody, long-acting monoclonal antibody, and maternal vaccine would have incremental cost-effectiveness ratios of $4280 (95% CI $1892 to $122,434), $1656 (95% CI $734 to $9091), and $8020 (95% CI $3501 to $47,047) per disability-adjusted life-year averted, respectively.

**Conclusions and Relevance:**

In Mali, long-acting monoclonal antibody is likely to be cost-effective from both the government and donor perspectives at $3 per dose. Maternal vaccine would need higher efficacy over that measured by a recent trial in order to be considered cost-effective.

## Highlights

• New RSV prevention products can substantially reduce disease burden.
• Longer-acting monoclonal antibodies, priced affordably, are likely cost-effective.
• Maternal vaccines meeting preferred product characteristics would be cost-effective.
• RSV prevention products can provide good value in low-income countries.

## 1 Introduction

Respiratory syncytial virus (RSV) is the leading cause of acute lower respiratory tract infections (LRTI) in infants globally [1]. An estimated 93% of RSV-LRTI cases and 99% of RSV-LRTI deaths occur in low- and middle-income countries (LMICs) [2]. Children under six months of age account for approximately 45% of severe RSV cases [2].

The only product licensed for RSV-LRTI prevention is palivizumab—a humanized monoclonal antibody administered monthly during the RSV season to children at elevated risk for severe disease [3]. The palivizumab price point renders it cost-prohibitive for most LMICs. New monoclonal antibodies and vaccines against RSV-LRTI are in development. One dose of an extended half-life monoclonal antibody candidate, nirsevimab, may protect healthy preterm infants against RSV-LRTI for five months, covering the duration of a typical RSV season [4]. While an RSV fusion protein nanoparticle (RSV-F) vaccine candidate for pregnant women failed to meet its primary endpoint in a phase III clinical trial (subsequently referred to as RSV-F vaccine trial), secondary endpoints and subgroup analyses indicated that a single dose administered to pregnant women in their third trimester protected infants from clinically important RSV-LRTI outcomes over the first three months of life [5]. Given the advanced development of RSV-LRTI prevention candidates, it is important to consider the potential economic and health impacts these products could have in LMICs.

We have identified only one other cost-effectiveness analysis of RSV-LRTI prevention in LMICs [6]. This analysis relied on inputs from systematic reviews of global data and modelling data for disease incidence and severity. The Centre pour le Développement des Vaccins in Bamako, Mali has collected high quality RSV surveillance and cost of care data which allowed us the unique opportunity to conduct a country-specific cost-effectiveness analysis to inform policy and investment expectations for future RSV preventive interventions.

## 2 Methods

### 2.1 Model design

We modeled the costs and benefits of RSV-LRTI preventive interventions in Mali using a probability-based outcome tree with twelve simulated monthly birth cohorts followed through the first six months of life (Table 1, Supplement Fig. 1). Integrating parameter uncertainty, we estimated distributions of expected RSV-associated health and economic outcomes under four scenarios: a) status quo without intervention, b) intra-seasonal infant prophylaxis with monthly doses of short-acting monoclonal antibody (short-acting mAb), c) pre-seasonal infant prophylaxis with a single birth dose of long-acting monoclonal antibody (long-acting mAb), and d) year-round, single dose maternal vaccination. With these outcome sets, we calculated the incremental cost-effectiveness ratio for each product at specific price points and the maximum product cost given a specific cost-effectiveness threshold.

### 2.2 Heath outcomes

The health outcomes of interest were the expected number of RSV-LRTI cases, hospitalizations, and deaths for each birth cohort during the first six months of life. We derived the incidence of RSV-LRTI under status quo from a community-based study of RSV incidence in Bamako, Mali [7], which was nested within a maternal influenza vaccine clinical trial [8]. We calculated probabilities of RSV illness dependent on calendar month and infant age (Supplement Fig. 2). Given laboratory-confirmed RSV illness, probabilities of RSV-LRTI and hospitalization were based on observations in the community-based study (Table 1). RSV-LRTI was defined as clinical pneumonia using World Health Organization (WHO) criteria [8]. Due to the low frequency of RSV-associated deaths observed in the influenza vaccine trial, we obtained Mali-specific, RSV-attributable mortality estimates from the Pneumonia Etiology Research for Child Health (PERCH) study among hospitalized children (C. Prosperi, personal communication, September 7, 2020) [1].

**Table 1:** Summary of input parameters used for the base case analysis.

	Value (95% Confidence Interval, when used)	Study setting	Rationale
**Epidemiologic parameters**			
RSV incidence rate (per 1,000 person-years), by month of life		Mali	Age-specific community-based incidence rates per 1000 person-years in Mali [7]. Incidence varies by infant age as well as calendar month.
*Month 1*	141.6 (70.1, 229.7)		
*Month 2*	208.7 (119.8, 320.0)		
*Month 3*	539.5 (386.9, 713.9)		
*Month 4*	575.7 (408.9, 763.9)		
*Month 5*	1046.8 (817.5, 1290.8)		
*Month 6*	996.9 (736.7, 1292.6)		
Probability of LRTI given RSV	0.13 (0.11, 0.17)	Mali	All infants with pneumonia episodes occurring between October 2012 and May 2013 were selected to be tested for RSV in the Mali incidence study, whereas only 48.9% of the infants with influenza-like-illness without pneumonia were tested [7]. To account for the oversampling among infants with pneumonia, we calculate the probability of LRTI given RSV as the proportion of RSV cases with pneumonia reported in the trial adjusted to match the proportion of infants with influenza-like-illness but without pneumonia who were tested for RSV.
Probability of inpatient care given RSV-LRTI	0.29 (0.20, 0.37)	Mali	Among the proportion of infants less than six months with confirmed RSV-pneumonia in the RSV incidence study in Mali, 29% required inpatient care [7]. All infants with pneumonia who did not receive inpatient care received outpatient care. The probability of outpatient care given RSV-LRTI is calculated as 1- probability of inpatient care given RSV-LRTI.
Case fatality rate among those who received inpatient care given RSV-LRTI	0.016 (0.0038, 0.037)	Mali	The case fatality rate for infants less than six months with RSV-pneumonia who received inpatient care observed in the Pneumonia Etiology Research for Child Health site in Mali [1]. This case fatality rate was calculated by C. Prosperi using a Bayesian integrated approach to adjust for the RSV etiologic fraction (personal communication, September 7, 2020) [25].
Disability weight inpatient RSV-LRTI	0.13 (0.10, 0.17)	Multi-national	The 2017 Institute for Health Metrics and Evaluation Global Burden of Disease disability weight for an acute episode of a severe LRTI, used to calculate DALYs [26].
Disability weight outpatient RSV-LRTI	0.05 (0.04, 0.07)	Multi-national	The 2017 Institute for Health Metrics and Evaluation Global Burden of Disease disability weight for an acute episode of a moderate LRTI, used to calculate DALYs [26].
Duration of RSV illness (days)	8.5 (7, 10)	Multi-national	The average duration of illness for episodes of lower-respiratory tract infections based on health facility data [27].
**Demographic parameters**			
Crude birth rate (per 1,000)	42	Mali	The World Bank crude birth rate per 1,000 total population for Mali in 2017 [16].
Total country population	18,540,000	Mali	The World Bank total population estimate for Mali in 2017 [16].
Number of infants in each birth cohort	59,734 – 66,134	Mali	The number of infants in each monthly birth cohort was calculated by first multiplying the crude birth rate by the total country population for Mali to estimate the number of infants born in one year. The total number of births for each month was assigned based on the number of days in each month.
Life expectancy at birth	58	Mali	Average 2017 life expectancy at birth for Mali, used to calculate DALYs [16].
**Economic parameters**			
GDP per capita (USD)	891	Mali	The Gross Domestic Product (GDP) per capita has been previously recommended by the WHO as a willingness-to-pay threshold for economic analyses in low- and middle-income countries. We used the World Bank reported GDP per capita for Mali in 2019 [16].
Inpatient care costs (USD)	118.57 (92.20, 144.68)	Mali	Average cost of medical care for infants less than six months with confirmed RSV illness who received inpatient care. Costs are inclusive of outpatient services also acquired by this group [11].
Outpatient care costs (USD)	6.56 (5.44, 7.66)	Mali	Average cost of medical care for infants less than six months with confirmed RSV illness who received outpatient care services only [11].
Intervention delivery cost per dose (USD)	1.35	Low-income countries	Average cost of adding one product to existing immunization programs in low-income countries in 2019 US dollars [12].
**RSV Intervention parameters**			
Short-acting mAb efficacy	0.78 (0.60, 0.90)	North America, United Kingdom	Based on the multinational phase III Impact-RSV prevention trial, monthly prophylaxis with palivizumab results in a 78% reduction of RSV hospitalizations among pre-term infants without bronchopulmonary dysplasia [3]. Without access to the same life-saving postnatal interventions that are available in high-income countries (such as supplemental oxygen and mechanical ventilation), infants born with bronchopulmonary dysplasia in low-income countries do not often survive past the neonatal period, and therefore we used efficacy data for palivizumab which excluded this high-risk group. Although palivizumab has never been evaluated in healthy term infants, a similar monoclonal antibody, motavizumab, was shown to reduce RSV hospitalizations in this group [28].
Long-acting mAb efficacy	0.70 (0.52, 0.81)	Multi-national	Study results from a multinational phase II clinical trial for nirsevimab in healthy preterm infants between 29- and 35-weeks gestational age and entering their first RSV season indicate a 70.1% reduction in medically attended RSV confirmed LRTI for infants receiving a single 50 mg dose of nirsevimab compared to placebo [4].
Maternal vaccine efficacy	0.56 (0.36, 0.70)	South Africa	Results from infants enrolled at the South African clinical sites in the Novavax Phase III Prepare™ Trial demonstrate a 56% reduction in medically attended RSV-LRTI [5].
Short-acting mAb duration of protection (months)	1	North America, United Kingdom	A single intramuscular 15 mg/kg bodyweight dose of palivizumab provides one month of protection against RSV. Doses are administered to infants in 30-day intervals throughout the RSV season [3].
Long-acting mAb duration of protection (months)	5	Multi-national	Nirsevimab has an extended serum half-life compared to previous anti-RSV monoclonal antibodies. A single 50 mg intramuscular dose of nirsevimab can provide up to five months of protection [4].
Maternal vaccine duration of protection (months)	3	Multi-national	RSV-F vaccine was administered to pregnant mothers in their third trimester and can protect newborns from RSV disease for up to three months post-birth [5].
Short-acting mAb coverage	77.0%	Mali	The WHO and UNICEF estimate for 2018 DTP3 vaccine coverage in Mali is used as a proxy for short-acting monoclonal antibody coverage over multiple doses [29].
Long-acting mAb coverage	83.0%	Mali	The WHO and UNICEF estimate for 2018 BCG immunization coverage in Mali is used as a proxy for a single birth dose of long-acting monoclonal antibody [29].
Maternal vaccine coverage	35.5%	Mali	To determine the probability of a pregnant woman receiving the maternal vaccine, we multiplied the proportion of women attending at least four antenatal care visits (ANC4 + ) by vaccination coverage for the first dose of diphtheria-tetanus-pertussis vaccine (DTP1), as has been done previously [30]. The UNICEF estimate for 2018 ANC4 + in Mali is 43.3% [31]. The WHO and UNCIEF estimate for 2018 DTP1 coverage is 82% [29].

We measured health impact in disability-adjusted life-years (DALYs), a metric that combines life-years lost to premature death with productive life-years lost to illness and disability (Online Supplement) [9]. Years of life lost were discounted at 3% annually [10].

### 2.3 Interventions

We parameterized intervention efficacy and durability based on characteristics of tested products (Table 1). The efficacy and duration of protection for the products were 78% over one month for short-acting mAb [3], 70% over five months for long-acting mAb [4], and 56% over three months for maternal vaccine [5]. We applied maternal vaccine characteristics specific to a South African subgroup from the RSV-F vaccine trial [5]. Although the trial included multiple sites, South Africa was the only site in Africa and had the closest RSV-LRTI incidence to Mali. We applied product-specific administration schedules (Supplement Fig. 3). Both short-acting mAb and long-acting mAb were administered only when the duration of protection coincided with the RSV season (Online Supplement). Pregnant mothers in their third trimester were eligible for a single dose of maternal vaccine at any time of year. We projected coverage using 2018 Mali-specific data for routine immunization or antenatal care (Table 1, Online Supplement). We also explored the product health impact at a range of alternative coverage estimates spanning 0 to 100%.

### 2.4 Economic outcomes

We derived medical and hospitalization costs from a 2013 cost-effectiveness study conducted as part of the Mali maternal influenza vaccine clinical trial (Table 1, Online Supplement) [11]. For both mAbs and maternal vaccine, we assumed an administration cost per dose of $1.35, reflecting the incremental cost of adding one product to the Mali national immunization schedule [12].

### 2.5 Budget impact and cost-effectiveness

We delineated four perspectives for evaluating budget impact and cost-effectiveness: household, government, donor, and societal. Households bear the economic cost of medical care for RSV illness but would not bear any cost of intervention. The Malian government would bear the costs of delivery and product administration [13]. We assumed Gavi, the Vaccine Alliance, would be the donor organization bearing costs of vaccine procurement. Like other LMICs in sub-Saharan Africa, Mali receives support from Gavi for much of its national immunization program. The Malian government would co-finance $0.20 per product dose [14], and the remaining price would be paid by Gavi. Therefore, the government perspective includes administration costs plus a consistent co-financing contribution regardless of product price, while the donor perspective is conditional on price. The societal perspective considers all costs regardless of payer.

We calculated the budget impact for each intervention as the expected change in spending due to medical or intervention costs compared to status quo over a single year and delineated by perspective. The incremental cost-effectiveness ratio (ICER) for each intervention compared to the status quo was calculated as Δc/Δe, where Δc is the difference in economic costs between intervention and status quo, and Δe is the change in health outcomes. We did not conduct a head-to-head comparison of products, as the final assortment of choices available to countries is not yet certain.

We integrated parameter uncertainty into our analysis using a Monte Carlo approach with 10,000 independent trials. For each trial, one value was randomly sampled from every distribution of probabilities and costs. We then calculated the health and economic outcomes associated with status quo and each intervention, holding parameters constant across each arm. The 95% confidence intervals for any given outcome were then defined as the range encompassing 95% of the values produced across all trials. The point estimate reported for each outcome is the value produced by executing the simulation using the point estimate specified for each input parameter.

For evaluating the probability that an intervention would be cost-effective across a range of values for willingness-to-pay (WTP) for DALYs, we applied a net health benefits framework [15]. WTP indicates the maximum spending acceptable to avert one DALY. Net health benefits are calculated as: Δe – (Δc/WTP). Under this approach, any intervention that results in positive net health benefits is considered cost-effective [15]. We evaluated the interventions across a range of WTP values spanning from $0 to $20,000 per DALY. At any specific WTP, we calculated the probability that an intervention would be cost-effective as the proportion of trials with positive net health benefits. For illustrative purposes, we use a WTP of $891 per DALY [10], equivalent to the 2019 per-capita Gross Domestic Product in Mali [16].

### 2.6 Sensitivity analysis

We performed a series of univariate analyses to identify which parameters would have the greatest influence on the results. We varied each individual parameter between its lower and upper 95% confidence limits, with all other parameters held at their point estimate. We then recorded the incremental cost-effectiveness ratio for each product at each limit.

### 2.7 Secondary analyses

We conducted six secondary analyses to assess the sensitivity of our conclusions to changes in model structure and assumptions (Table 2). For each, we altered the relevant feature while retaining all other model elements, including the Monte Carlo sampling. We evaluated the cost-effectiveness of the following: 1) maternal vaccine meeting WHO preferences for efficacy and duration [17]; 2) maternal vaccine with the overall efficacy from the RSV vaccine trial rather than from South Africa subset data; 3) providing a long-acting mAb as a birth dose and within routine immunization schedules compared to birth dose alone; 4) pre-seasonal maternal vaccine administration, instead of year-round; 5) base case interventions assuming they prevent RSV Upper Respiratory Tract Infections (URTI) in addition to RSV-LRTI; and 6) base case interventions assuming all infants receive appropriate medical care.

**Table 2:** Secondary analyses and rationale.

Secondary analyses	Rationale
1. To assess the cost effectiveness of a hypothetical RSV maternal vaccine product meeting WHO recommendations for efficacy and duration	There is a robust pipeline of candidate RSV preventive interventions in development. This analysis uses WHO preferences for product efficacy inputs rather than published efficacy data for limited RSV maternal vaccine products to date. The WHO preferred product characteristics for RSV maternal vaccines are at least 70% efficacy from birth to age four months [17].
2. To assess cost-effectiveness of providing mAb as a birth dose and within routine immunization schedules compared to birth dose alone	Since coverage of birth dose mAb is not expected to be perfect, this analysis explores the potential product cost-effectiveness of an alternative delivery strategy which provides catchup mAb to children during routine immunization visits during the first year of life. Guided by age-specific RSV attack rates estimated for infants from birth to 12 months of age in low-income countries [2], we projected attack rates among infants aged six to 12 months in Mali by applying a linear decline over this period, starting with the attack rate among children aged six months and ending at the attack rate among children aged three months. We then identified the routine pediatric immunization visit at which an older infant should receive the long-acting mAb, based on birth month.
3. To assess the cost-effectiveness of a pre-seasonal RSV vaccination strategy	Pre-seasonal maternal RSV vaccination would decrease the product costs as compared to year-round strategies. This analysis explores the cost-effectiveness of pre-seasonal vaccination.
4. To assess cost-effectiveness of maternal vaccine using overall efficacy of RSV-F vaccine rather than subset data from South Africa	The maternal vaccine efficacy estimate used in the base case is from a subset analysis of a clinical trial. This analysis uses the overall efficacy estimate against medically attended RSV-LRTI from the clinical trial, 39.4% (95% CI 5.3% to 61.2%) as a model input instead [23].
5. To assess cost-effectiveness of interventions assuming they prevent RSV Upper Respiratory Tract Infections (URTI) in addition to LRTI	Future RSV preventive interventions may have activity against RSV-URTI in addition to prevention of RSV-LRTI. This analysis assesses the cost effectiveness of interventions that prevent both outcomes. RSV-URTI was captured in the community-based incidence study in Mali through active surveillance for febrile acute respiratory infection, defined in the parent trial as a child presenting with fever in combination with any of the following: runny nose, nasal congestion, cough, difficulty breathing, purulent drainage from ear, or wheezing [8]. Among infants meeting testing criteria and with confirmed RSV illness, we assumed all those without RSV-LRTI developed RSV-URTI (Supplement Figure 5). For infants with RSV-URTI in the study, outpatient care was provided to 93.6%, and all others received no medical care [7]. Disability weights used to calculate DALYs for infants with RSV-URTI were 0.05 (95% CI 0.04 to 0.07) for those who received outpatient care and 0.006 (95% CI 0.003 to 0.010) for infants who did not receive care [26]. We evaluated the cost-effectiveness of each intervention presuming any benefit to occur at the point of infection.
6. To assess cost-effectiveness of interventions assuming all infants with RSV-LRTI receive appropriate care	Among young children in LMICs, an estimated 53% of severe RSV-LRTI episodes do not receive inpatient care, and 49% of RSV-LRTI deaths occur outside the hospital [2]. This secondary analysis assumes all infants with RSV-LRTI who require inpatient care receive it, and that all deaths due to RSV-LRTI occur in inpatient care settings.

All analyses were performed using R 4.0.2 (https://r-project.org).

## 3 Results

**Fig. 1:**
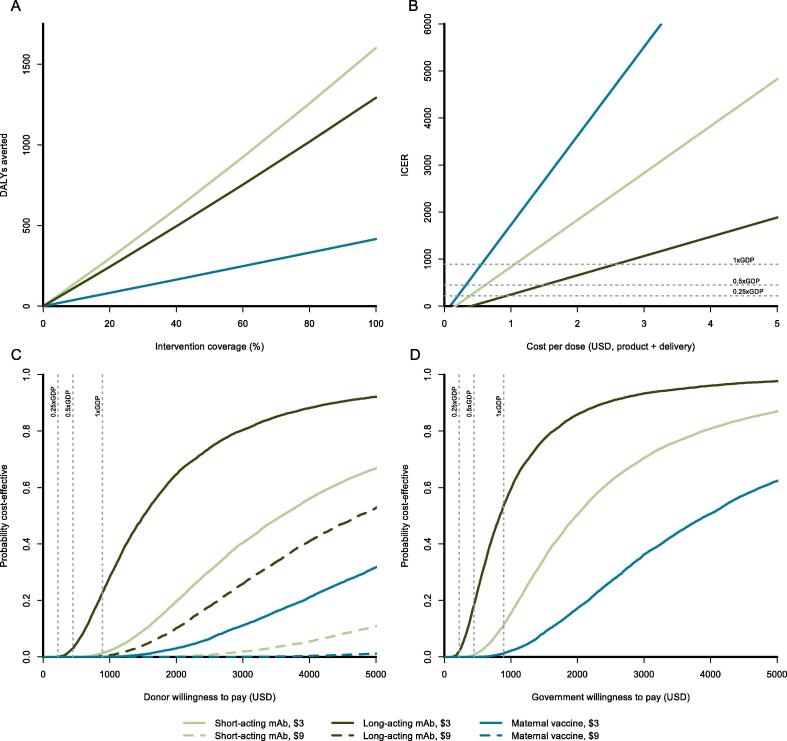
**A)** Disability-adjusted life-years (DALYs) averted as intervention coverage increases for a theoretical birth cohort of infants born in Mali followed for the first six months of life. **B)** Incremental cost-effectiveness ratio (ICER) as cost per full administration increases for each intervention, from the societal perspective. **C)** Probability that each intervention would be considered cost-effective from a donor perspective at a given willingness-to-pay for disability DALYs, and at price points of $3 or $9 per dose in 2019 United States dollars (USD). **D)** Probability that each intervention would be considered cost effective at a given willingness-to-pay for DALYs, from a government perspective. The gray dotted lines indicate the willingness-to-pay (WTP) thresholds, equal to the per capita gross domestic product (1xGDP) for Mali, 0.5xGDP, and 0.25xGDP, as labeled. An ICER less than the per capita GDP could be considered “very cost-effective” by former WHO standards.

We estimated the DALY loss associated with RSV-LRTI for a one-year birth cohort in Mali followed until six months of age under status quo and preventative interventions. For our base case, we estimated that short-acting mAb, long-acting mAb, and maternal vaccine could avert 1206 (95% CI 627 to 7211) DALYs, 1059 (95% CI 201 to 2354) DALYs, and 146 (95% CI 26 to 331) DALYs, respectively. The health impact of each intervention is sensitive to changes in coverage rate (Fig. 1A, Supplement Table 2). If each intervention had 50% coverage, short-acting mAb, long-acting mAb, and maternal vaccine could avert 762 DALYs (95% CI 147 to 1727), 623 DALYs (95% CI 117 to 1404), and 206 DALYs (95% CI 35 to 472), respectively (Fig. 1A). At a product cost of $3 and delivery cost of $1.35 per dose, short-acting mAb, long-acting mAb, and maternal vaccine would carry intervention costs of $3,796,230, $1,560,246, and $2,184,439 respectively, while averting $123,726, $101,165, and $33,492 in medical costs (Table 3). From the Malian government perspective, the total annual budget impact of these interventions would be $1,208,498, $404,484, and $602,598 for short-acting mAb, long-acting mAb, and maternal vaccine, respectively (Table 3).

**Table 3:** Budget impact analysis for adopting each intervention at a product cost of $3 per dose, compared to status quo. Costs of RSV care and prevention are delineated by payer and expressed in 2019 US dollars. Costs represent those applicable for a single year-long birth cohort, with infants followed to six months of life. Calculations of Incremental cost-effectiveness ratios in the table may not match exactly due to rounding.

	Household perspective	Government perspective	Donor perspective	Societal perspective
Cost (95% CI), USD				
Status quo costs	525,181 (294,999; 695,323)	NA	NA	525,181 (294,999; 695,323)
Short-acting mAb costs	− 195,647 (107,188; 263,466)	+ 1,861,086	+ 3,366,867	+ 5,032,307
Long-acting mAb costs	− 171,909 (89,729; 263,466)	+ 671,443	+ 1,214,698	+ 1,714,231
Maternal vaccine costs	− 23,731 (10,710; 33,593)	+ 427,917	+ 774,139	+ 1,178,325
DALYs averted (95% CI)				
Short-acting mAb	1206 (225; 2689)	1206 (225; 2689)	1206 (225; 2689)	1206 (225; 2689)
Long-acting mAb	1059 (191; 2345)	1059 (191; 2345)	1059 (191; 2345)	1059 (191; 2345)
Maternal vaccine	146 (25; 328)	146 (25; 328)	146 (25; 328)	146 (25; 328)
Incremental Cost-Effectiveness Ratio (USD per DALY averted)				
Short-acting mAb	Cost-saving	1544	2793	4164
Long-acting mAb	Cost-saving	634	1147	1614
Maternal vaccine	Cost-saving	2926	5294	8038

**Fig. 2:**
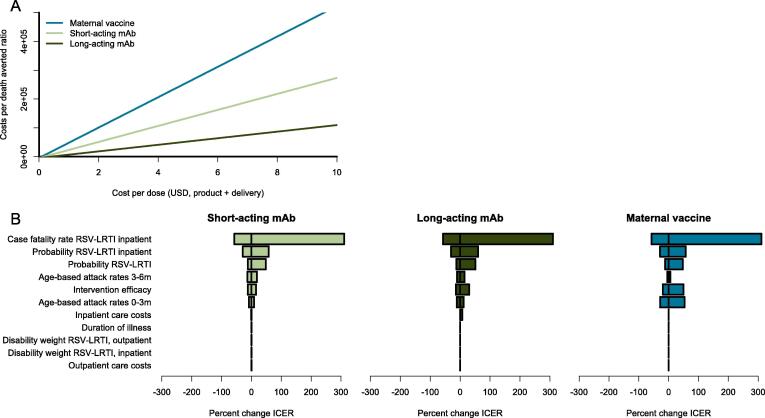
**A)** Cost per death averted ratio as total intervention costs are varied from $0 to $10 per dose. **B)** Tornado plot of parameter variance influence on the incremental cost-effectiveness ratio (ICER) for each intervention, priced at $3 per dose. Parameters with greater impact on the ICER have larger bars.

From the societal perspective, the ICER associated with each intervention would be $4280 (95% CI $1892 to $122,434), $1656 (95% CI $734 to $9091), and $8020 (95% CI $3501 to $47,047) per DALY averted, respectively (Fig. 1B). The cost per death averted for short-acting mAb, long-acting mAb, and maternal vaccine would be $116,342, $4,5104, and $224,593 for these interventions, respectively (Fig. 2A). All ICER values exist in quadrant 1 of the cost-effectiveness plane.

We evaluated the relationship between WTP for a DALY and intervention cost-effectiveness from the donor and government perspectives independently. At a product price of $3 per dose, long-acting mAb is preferable with greater than 50% likelihood of being cost-effective from the donor perspective at WTP above $1521 per DALY (Fig. 1C). For short-acting mAb and maternal vaccine, this mark is achieved at WTP values of $3638 and $7118, respectively. At a product price of $9 per dose, long-acting mAb and short-acting mAb are preferable from the donor perspective at WTP above $4781, $11,435 per DALY, respectively. Maternal vaccine is less than 50% likely to be cost-effective at a product price of $9 and WTP of $20,000. From the government perspective, where vaccine program costs are not influenced by product price, long-acting mAb is preferable at WTP above $841 per DALY (Fig. 1D). For short-acting mAb and maternal vaccine, these WTP values are $2011 and $3934, respectively.

We conducted a series of one-way sensitivity analyses to identify the parameters whose variance had the largest influence on the ICER. The most influential parameter across interventions was the inpatient case fatality rate, capable of modifying the ICER by up to 315% over the point estimate (Fig. 2B). Parameters with less influence—where the ICER remained within less than a 60% change from the point estimate—included the probability of receiving inpatient care, probability of LRTI given RSV, age-based RSV attack rates, intervention product efficacy, and inpatient care costs. For all other parameters, the ICER remained within less than a one percent change from the point estimate.

### 3.1 Secondary analyses

**Fig. 3:**
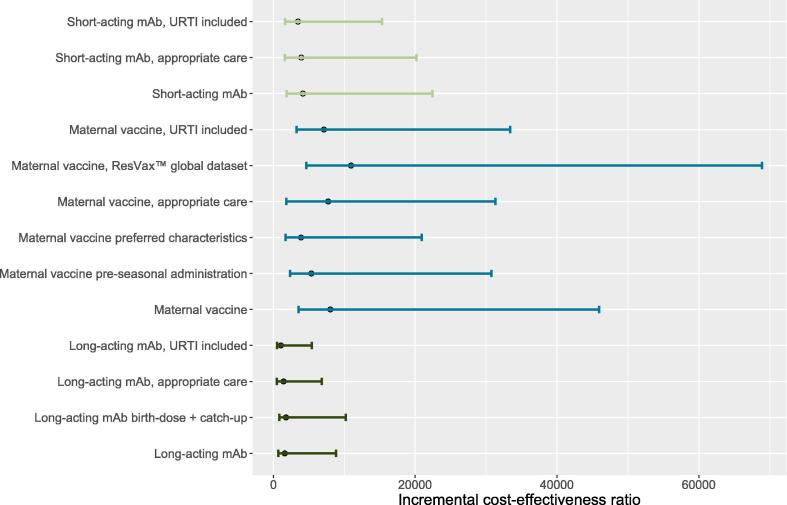
The incremental cost-effectiveness ratio for each intervention scenario from the societal perspective. Filled circles represent the point estimate and error bars indicate 95% credible intervals.

We conducted six secondary analyses to assess changes in model structure and assumptions (Fig. 3).

1. If maternal vaccine efficacy and duration were replaced with the WHO preferred product characteristics for a maternal RSV vaccine, the vaccine health impact would increase by 102%. A maternal vaccine meeting WHO preferred efficacy and duration would have an ICER of $3901 (95% CI $1748 to $21,169) per DALY averted.
2. If maternal vaccine efficacy were replaced with overall RSV-F vaccine trial results instead of South Africa site-specific results, product efficacy would decrease and the ICER would increase to $10,946 (95% CI $4707 to $69,446) per DALY averted.
3. If long-acting mAb were also given during a scheduled routine pediatric immunization visit to older infants who would have been ineligible for the seasonal birth dose based on their birth month, more infants would be protected during the RSV season. However, RSV mortality rates are lower for these older infants, so the ICER would increase to $1783 (95% CI $852 to $9618) per DALY averted.
4. If maternal vaccine were given to pregnant women during a pre-seasonal campaign instead of year-round, then the ICER would decrease to $5354 (95% CI $2351 to $31,523) per DALY averted.
5. If the model included RSV-URTI health and economic outcomes, then health impact would increase for each intervention. Short-acting mAb, long-acting mAb, and maternal vaccine would have ICERs of $3475 (95% CI $1657 to $15,582), $1071 (95% CI $543 to $5473), and $7127 (95% CI $3259 to $34,678) per DALY averted, respectively.
6. If all infants with RSV-LRTI were to receive appropriate medical care, then the costs associated with RSV-LRTI illness would be higher than base case estimates. Short-acting mAb, long-acting mAb, and maternal vaccine would have ICERs of $3938 (95% CI $1638 to $20,365), $1441 (95% CI $479 to $7067), and $7733 (95% CI $1858 to $32,634) per DALY averted, respectively.

## 4 Discussion

We estimated that if short-acting mAb (intra-seasonal), long-acting mAb (pre-seasonal birth dose), or maternal vaccine (year-round) programs were implemented in Mali, the incremental cost to society would be $4280, $1656, and $8020 per DALY averted, respectively. This indicates that long-acting mAb may provide better value than either short-acting mAb or maternal vaccine in Mali and similar low-income countries. We evaluated the probability of each intervention being cost-effective across a broad range of willingness-to-pay values from government and donor perspectives. From the government perspective, implementing long-acting mAb is likely to be cost-effective at willingness-to-pay values approaching the per capita GDP of Mali. The per capita GDP has been a commonly used threshold for health intervention cost-effectiveness [10]. Recent studies examining country-level health spending have suggested that the actual willingness or ability to pay for health in Mali may be much lower, in the range of $14 to $311 per DALY [18], [19]. Mali is one of the lowest-income countries in the world, and therefore donors considering a global portfolio may elect to support RSV prevention due to more favorable economic considerations in other countries. Therefore, if these interventions are supported by global donors, Mali may need subsidies to offset the administrative costs of RSV prevention as well as the product price. Ultimately, the decision about whether the benefits of investment outweigh the costs lies with the Malian people and their government.

In addition to providing sufficient value for money, new health interventions must be affordable [20]. The annual budget impact of adding long-acting mAb to the current immunization program in Mali would be a 0.21% increase of the overall 2017 health budget [16]. Gavi is the likely donor for RSV prevention products in Mali and other LMICs. Gavi has indicated support for such products, contingent on regulatory approvals and value for money commensurate with its investment case [21]. Our estimation of cost per death averted by long-acting mAb at $3 per dose, similar to the price negotiated by Gavi for a single dose of 13-valent pneumococcal conjugate vaccine [22], falls within the range for the Gavi investment case. While many of our parameters differ from those used in the investment case, the lower Mali-specific RSV-LRTI case fatality rate used in our analysis is balanced by the high observed attack rate.

In our secondary analyses, we assessed multiple alternative delivery and product performance scenarios. Our base case findings were generally conservative, and the alternatives led to lower incremental cost-effectiveness ratios for all interventions. In practical terms, if one or more of these alternative scenarios were true, then the RSV preventive interventions would more likely be cost-effective. As the exception, replacing the South African site-specific vaccine efficacy results from the RSV-F vaccine trial with overall efficacy results would raise the incremental cost-effectiveness ratio. In that trial, the overall 39.4% efficacy (95% CI 5.3 to 61.2%) against the primary endpoint, medically significant RSV-LRTI up to 90 days of life, did not achieve statistical significance [23]. There were significant reductions in a secondary endpoint, RSV-LRTI with severe hypoxia, as well as RSV-LRTI exploratory endpoints over the same duration of follow up, establishing proof-of-principle for maternal RSV vaccination to prevent medically important outcomes [23]. There is a robust pipeline of maternal RSV vaccines under development [17]. Our analysis indicates that a maternal vaccine with 70% efficacy, meeting WHO preferred product characteristics, would have a more favorable ICER. In addition to the challenges of product development and licensure, maternal immunization platforms in Mali and most LMICs require strengthening [24]. Donor support may therefore be required to implement a maternal vaccine program, but such investment could strengthen antenatal care health systems, providing broader benefits to maternal and child health overall.

The greatest strength of our study is use of country-specific RSV epidemiological and cost information. We used RSV incidence estimates from household surveillance standardizing identification of community cases. Nevertheless, our RSV incidence estimates came from a single full year of surveillance in urban Bamako, which were high compared to incidence estimates elsewhere [2]. We do not know whether the burden of RSV disease measured in Bamako reflected typically high disease burden in all of Mali or if it reflected an anomalous year at the study site. A previous cost-effectiveness analysis which assigned a lower burden of disease to Mali found that RSV preventive interventions would not be cost-effective unless the willingness-to-pay were as high as $2500 per DALY, despite using more favorable characteristics of the maternal vaccine [6], underscoring the influence of disease burden on cost-effectiveness. Another limitation is that our analysis ends at six months of age. To the extent that RSV disease may be deferred but not averted by short-acting products, we may overestimate the value of prevention. However, as the most severe disease is concentrated in the first six months of life [2], a focus on this period is warranted. Finally, neither long-acting mAb nor maternal vaccine products have achieved licensure. As new clinical data emerge for these and similar products, their cost-effectiveness should be updated.

While RSV-LRTI is a high burden disease, the only licensed RSV-LRTI preventive intervention is cost-prohibitive for most LMICs. Future long-acting mAb and maternal vaccines have the potential to address this unmet global health need [4], [23]. We used Mali-specific epidemiology and cost data to conduct cost-effectiveness analyses of three potential RSV interventions, short-acting mAb, long-acting mAb, and maternal vaccines. The long-acting mAb product currently in development is likely to be cost-effective at prices near to what Gavi pays for similar interventions, and maternal vaccines which meet WHO preferred product characteristics could also be cost-effective. Ultimately, global health will benefit from the availability of multiple RSV preventive interventions. While the licensed product performance characteristics, prices, and delivery costs will drive policy and investment decisions in LMICs, our work highlights the potential benefit that RSV preventive interventions can have in Mali and similar countries.

## Disclaimer

The authors alone are responsible for the views expressed in this publication, and they do not necessarily represent the decisions, policy, or views of their institutions.

## Funding

This work was financially supported by grant 1,088,264 from the Bill and Melinda Gates Foundation. The funding source had no role in the study design; in the collection, analysis, and interpretation of data; in the writing of the report; or in the decision to submit the article for publication.

## Data sharing

Model code is available at https://github.com/MCFitz/RSV-CEA-Mali

## Declaration of Competing Interest

The authors declare that they have no known competing financial interests or personal relationships that could have appeared to influence the work reported in this paper.
