# Supplementary material for: Cost-effectiveness of infant respiratory syncytial virus preventive interventions in Mali: A modeling study to inform policy and investment decisions
Source: Vaccine. 2021 Aug 16;39(35):5037–45. doi: 10.1016/j.vaccine.2021.06.086 (PMC8377743; doi:10.1016/j.vaccine.2021.06.086)
Supplement: Supplementary data 1 [file mmc1.docx]

Online Supplement

Cost-effectiveness of infant respiratory syncytial virus preventive interventions in Mali: a modelling study to inform policy and investment decisions

**Additional method details**

*Calculating disability-adjusted life-years (DALYs)*

For each mortality event, we calculated the years of life lost as the difference between 58 years, the 2017 life expectancy in Mali [1], and the age of death in months. We assigned DALY values for inpatient and outpatient RSV-LRTI based on a 2017 global burden of disease study (Table 1) [2].

*Seasonal product administration*

Over 70% of RSV cases in the Malian community-based study occurred during August and September [3]. As RSV season timing may shift, we extended the season to July through October, typical for other countries in the region [4]. In our model, infants younger than six months of age and entering their first RSV season were eligible for short-acting mAb, infants born during or within five months preceding the RSV season were eligible for a birth dose of long-acting mAb.

*Intervention coverage*

For short-acting mAb, we used 77.0%, the coverage for third dose of diphtheria-tetanus-pertussis immunization (DTP3) [5]. For long-acting mAb, we used 83.0%, the coverage for birth dose of bacille Calmette-Guérin immunization (BCG) [5]. For maternal immunization coverage, we used 35.5% based on a previous study which estimated maternal influenza immunization coverage in LMICs as the product of the percentage of women attending at least four antenatal care visits (ANC4) multiplied by the coverage of first dose of diphtheria-tetanus-pertussis immunization (DTP1) [6].

*Estimating RSV healthcare costs from Malian data*

We identified RSV positive specimens from the community-based RSV incidence study in Mali [3]. We matched them to corresponding specimens associated with healthcare cost data collected during the maternal influenza vaccine clinical trial, in which the RSV study was nested [7]. We subset these RSV cases into three categories: 1) those with any inpatient care costs, 2) those with costs but not for inpatient care, and 3) those with no associated costs. We converted medical costs back to their original currency, West African CFA francs, and then updated to 2019 CFA using an annual inflation rate of 0.155%, the average for Mali across 2013 to 2019 [8]. Finally, we transformed these costs to 2019 US dollars (USD), applying the average exchange rate for that year [8]. We recorded the mean and standard error of the total costs for infants who received inpatient care and the mean and standard error of the total costs for infants who received only outpatient care. These parameters defined the sampling distributions for the cost-effectiveness model.

**Additional Tables and Figures**

**Supplement Table 1.**Parameter sampling distributions.

|  | **Distribution** |
| --- | --- |
| RSV incidence rate (per 1,000 person-years), by month of life:  *Month 1*  *Month 2*  *Month 3*  *Month 4*  *Month 5*  *Month 6* | Beta (8, 670)  Beta (11, 621.4)  Beta (28, 594.8)  Beta (27, 535.8)  Beta (47, 491.8)  Beta (32, 353.2) |
| RSV positivity given influenza-like illness (θ) | Beta (110, 269) |
| Probability of LRTI given RSV | Beta (43, 110 + 839θ) |
| Probability of inpatient care given RSV-LRTI | Beta (13, 30) |
| Fatality rate of inpatient RSV-LRTI cases | Beta (2.4, 140.6) |
| Disability weight inpatient RSV-LRTI | Triangular (0.088, 0.190, 0.133) |
| Disability weight outpatient RSV-LRTI | Triangular (0.032, 0.074, 0.051) |
| Duration of RSV illness (days) | Normal (8.5, 0.77)^±^ |
| Inpatient care costs (USD) | Normal (118.57, 15.90) |
| Outpatient care costs (USD) | Normal (6.56, 0.67) |
| Short-acting mAb efficacy | Normal (0.78, 0.61)^±^ |
| Long-acting mAb efficacy | Log-normal (2.2, 0.76) |
| Maternal vaccine efficacy | Log-normal (1.82, 0.79) |

Distributions specified with the following parameters unless otherwise noted: Normal (mean, standard error); Log-normal (mean log, standard deviation log); Beta (alpha, beta), Triangle (lower limit, upper limit, mode)

^±^ Normal (mean, standard deviation)

**Supplement Table 2.** Intervention impact on health outcomes, including RSV cases, hospitalizations, deaths, and disability-adjusted life-years (DALYs).

|  | RSV cases | Hospitalizations | Deaths | DALYs |
| --- | --- | --- | --- | --- |
| Status quo | 201,524 (178,808; 224,961) | 3483 (1790; 4715) | 116 (22; 260) | 3236 (627; 7211) |
| Short-acting mAb | -75,074 (62,749; 88,504) | -1298 (554; 1582) | -43 (7; 85) | -1206 (231; 2695) |
| Long-acting mAb | -65,965 (50,047; 79,476) | -1140 (658; 1788) | -38 (8; 97) | -1059 (201; 2354) |
| Maternal vaccine | -9106 (5713; 12,369) | -157 (68; 232) | -5 (1; 12) | -146 (26; 331) |

**Supplement Figure 1.** Outcome tree model for theoretical birth cohorts of Malian infants followed from birth to six months of age. Ellipses indicate symmetrical sub-trees in the intervention arms.

**Supplement Figure 2.**Monthly RSV attack rates applied to theoretical birth cohorts of Malian infants from birth to six months of age. Color intensity is greatest in months with higher attack rates.

**Supplement Figure 3.** Product administration and duration of protection schedule for birth cohorts followed through six months of age. Months highlighted in peach indicate a typical RSV season for the region. Each cell represents a cohort of infants in a single month. For example, the row indicated by the arrow follows a cohort of infants born in April through six months of age. Short-acting mAb is provided to infants in multiple doses throughout the RSV season with one month of protection conferred per dose, a long-acting monoclonal antibody is administered seasonally as a single birth dose providing five months of protection, and a maternal vaccine administered to mothers during their third trimester protects newborns for three months post-birth.

**Supplement Figure 4.** Comparison of the density distribution of case fatality rate inputs for RSV-LRTI inpatient cases based on data from community-based respiratory infection surveillance and the PERCH study site in Mali.

**Supplement Figure 5.** Alternative outcome tree adjusted to include a pathway for RSV-URTI. Ellipses indicate symmetrical sub-trees in the intervention arms.

**References:**

[1]        Mali Data 2020. https://data.worldbank.org/country/mali (accessed April 23, 2020).

[2]        Global Burden of Disease Study 2017 (GBD 2017) Disability Weights | GHDx n.d. http://ghdx.healthdata.org/record/ihme-data/gbd-2017-disability-weights (accessed April 30, 2020).

[3]        Buchwald AG, Tamboura B, Tennant SM, Haidara FC, Coulibaly F, Doumbia M, et al. Epidemiology, Risk Factors, and Outcomes of Respiratory Syncytial Virus Infections in Newborns in Bamako, Mali. Clin Infect Dis 2020;70:59–66. https://doi.org/10.1093/cid/ciz157 10.1093/cid/ciz157.

[4]        Li Y, Reeves RM, Wang X, Bassat Q, Brooks WA, Cohen C, et al. Global patterns in monthly activity of influenza virus, respiratory syncytial virus, parainfluenza virus, and metapneumovirus: a systematic analysis. The Lancet Global Health 2019;7:e1031–45. https://doi.org/10.1016/S2214-109X(19)30264-5.

[5]        WHO and UNICEF estimates of national immunization coverage n.d. https://www.who.int/immunization/monitoring_surveillance/data/mli.pdf?ua=1 (accessed May 9, 2020).

[6]        Debellut F, Hendrix N, Ortiz JR, Lambach P, Neuzil KM, Bhat N, et al. Forecasting demand for maternal influenza immunization in low- and lower-middle-income countries. PLoS ONE 2018;13. https://doi.org/10.1371/journal.pone.0199470.

[7]        Orenstein EW, Orenstein LA, Diarra K, Djiteye M, Sidibe D, Haidara FC, et al. Cost-effectiveness of maternal influenza immunization in Bamako, Mali: A decision analysis. PLoS One 2017;12:e0171499. https://doi.org/10.1371/journal.pone.0171499 10.1371/journal.pone.0171499. eCollection 2017.

[8]        IMF Data - By Country n.d. https://data.imf.org/?sk=85b51b5a-b74f-473a-be16-49f1786949b3 (accessed July 30, 2020).
